# Secreted HLA-Fc fusion profiles immunopeptidome in hypoxic PDAC and cellular senescence

**DOI:** 10.1093/pnasnexus/pgad400

**Published:** 2023-12-14

**Authors:** Nicholas J Rettko, Lisa L Kirkemo, James A Wells

**Affiliations:** Department of Pharmaceutical Chemistry, University of California SanFrancisco, San Francisco, CA 94158, USA; Department of Pharmaceutical Chemistry, University of California SanFrancisco, San Francisco, CA 94158, USA; Department of Pharmaceutical Chemistry, University of California SanFrancisco, San Francisco, CA 94158, USA; Department of Cellular and Molecular Pharmacology, University of California SanFrancisco, San Francisco, CA 94158, USA

**Keywords:** HLA, immunopeptidomics, hypoxia, senescence

## Abstract

Human leukocyte antigens (HLA) present peptides largely from intracellular proteins on cell surfaces. As these complexes can serve as biomarkers in disease, proper identification of peptides derived from disease-associated antigens and the corresponding presenting HLA is important for the design and execution of therapeutic strategies. Yet, current mass spectrometry methods for immunopeptidomic profiling require large and complex sample inputs, hindering the study of certain disease phenotypes and lowering confidence in peptide and allele identification. Here, we describe a secreted HLA (sHLA)-Fc fusion construct for simple single HLA allele profiling in hypoxic pancreatic ductal adenocarcinoma (PDAC) and cellular senescence. This method streamlines sample preparation, enables temporal control, and provides allele-restricted target identification. Over 30,000 unique HLA-associated peptides were identified across 2 different HLA alleles and 7 cell lines, with ∼9,300 peptides newly discovered. The sHLA-Fc fusion capture technology holds the potential to expedite immunopeptidomics and advance therapeutic interest in HLA-peptide complexes.

## Introduction

HLA class I molecules present peptides, largely from intracellular proteins, on the surface of somatic cells (1). This pool of HLA-associated peptides, the immunopeptidome, reflects the proteome of a cell in a given phenotype (2, 3). Hence, peptides derived from mutated oncoproteins or disease-associated antigens in HLA–peptide complexes can act as biomarkers and immunotherapeutic targets. Recent years have seen promising cytotoxic potential from antibodies or T-cell receptors targeting tumor-associated HLA–peptide complexes, such as those involving Wilms tumor protein (WT1) (4).

Mass spectrometry (LC-MS/MS) analysis is the most common way to broadly identify these peptides, but faces challenges, such as large sample input (5), cell adherence (6), and notably, pan-major histocompatibility complex (MHC) immunoprecipitation. The high polymorphism of HLA molecules and the potential for peptides to bind multiple HLA molecules (7) can complicate antigen identification and therapeutic development. Additionally, as HLA-associated peptides are inherently difficult to analyze due to similarity in size and amino acid composition (8), multiallelic peptide samples can further decrease confidence in peptide origin and sequence.

Engineered cell lines expressing single HLA constructs help circumvent some of these challenges. Membrane-bound monoallelic HLA cell lines like B721.221 have aided in identifying thousands of HLA-associated peptides, but their usage is confined to HLA-null cell lines, limiting the cell types and biological contexts that can be examined (7, 9). Others have developed soluble HLA constructs lacking the transmembrane domain (10), but these remain unoptimized and require large bioreactors and large amounts of material (10–25 mg), as it demands several downstream purification and fractionation steps (9, 11, 12). These constraints make current monoallelic methods unsuitable for identifying disease-specific immunopeptidomes.

Here, we describe a streamlined MS-based method based on a doxycycline-induced secreted HLA-Fc fusion construct (sHLA-Fc fusion). We applied this method to study how cell states such as hypoxia and cellular senescence affect the immunopeptidome. We show that the sHLA-Fc fusion method provides highly purified, temporally controlled, monoallelic samples without requiring cell lysis or peptide fractionation. Samples were prepared in a matter of hours as opposed to the 2–3 days required by typical immonopeptidomic methods (8). With samples ranging from 12 to 130 million cells, we identified >30,000 peptides across 7 cell lines and 2 HLA alleles ranging from ∼600 to 10,000 peptides per sample depending on cell line and condition. We identified unique phenotype-restricted HLA-associated peptides in both hypoxic and senescent cells. The sHLA-Fc fusion capture technology will accelerate the process of profiling the immunopeptidome in disease states and contribute to novel target discovery and therapeutic development.

## Results

**Fig. 1.**
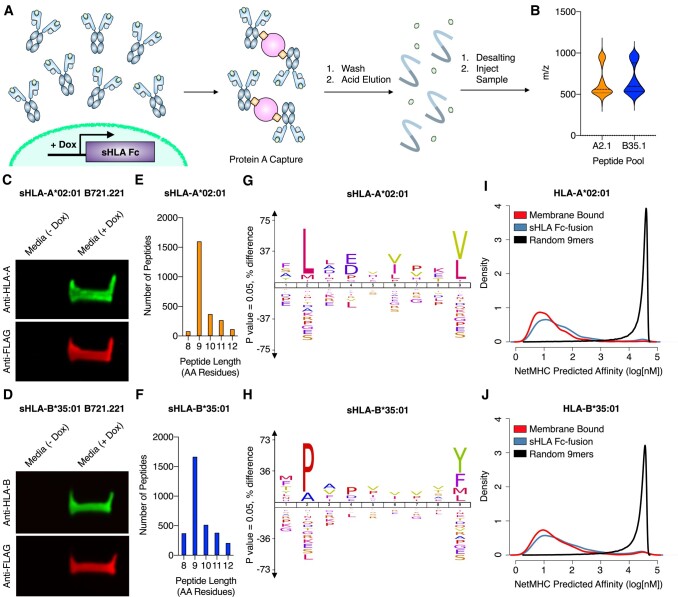
Workflow for sHLA cell line generation and subsequent immunopeptidomics. A) sHLA-Fc fusion is virally transduced into cells of interest. sHLA is induced using doxycycline after cells have undergone disease-specific perturbation. The media from the cells is isolated, sHLAs are immunoprecipitated and washed, and the peptides are eluted for analysis by LC-MS/MS. B) Mass:charge of identified peptides from sHLA monoallelic B721.221 cell lines (*n* = 2). C and D) Western blot of eluted sHLA-Fc fusion protein captured from doxycycline-treated or doxycycline-free media of sHLA monoallelic B721.221 cell lines expressing sHLA-A*02:01 or sHLA-B*35:01, respectively. E and F) Quantification of peptide length from cell lines expressing sHLA-A*02:01 or sHLA-B*35:01, respectively. G and H) ICE logos of 9-mer peptides from cell lines expressing sHLA-A*02:01 or sHLA-B*35:01, respectively. I and J) NetMHC predicted the affinities of the 9-mer peptides from sHLA-A*02:01 or sHLA-B*35:01 immunopeptidomics datasets, respectively, compared with published 9-mers identified from membrane-bound monoallelic B721.221 cells and a published list of 100,000 9-mer peptides (7, 9).

Fc fusion proteins have enhanced the expression, solubilization, and purification of extracellular membrane proteins (13, 14). As HLA proteins lacking the transmembrane domain have been shown to be properly folded and loaded with peptide cargo (11, 12), we hypothesized that a HLA-Fc fusion (henceforth referred to as sHLA) could be loaded and secreted similarly (Fig. 1A). With monoallelic B721.221 cell line datasets providing a standard dataset for peptide processing and binding prediction (7, 9), we sought to determine whether our sHLA-Fc fusion could capture HLA-associated peptides in this established cell model. We engineered two stable B721.221 cell lines, each transduced to express a sHLA-Fc fusion of either HLA-A*02:01 and HLA-B*35:01 under doxycycline induction. After 52 h with or without doxycycline treatment, media was collected and sHLA proteins were immunoprecipitated using magnetic protein A beads and analyzed by western blot (Fig. 1A and C). As the secretion of the sHLA-Fc fusion complex from B721.221 cells was successful, we subsequently isolated the sHLA-containing media from doxycycline-treated B721.221 cells and performed LC-MS/MS on the peptides derived from the sHLA-Fc fusion complexes through acid elution and standard desalting procedures. Data were analyzed using a stringent 1% FDR, and *m*/*z* spectra demonstrated clean +1 and +2 charge populations (Fig. 1B). The peptide search (PEAKS Online) identified consensus peptides in close agreement with peptide lengths (Fig. 1E and F), dominant anchor residues (Fig. 1G and H), and affinity prediction profiles (Fig. 1I and J) of previously reported membrane-bound HLA for each allele (9).

**Fig. 2.**
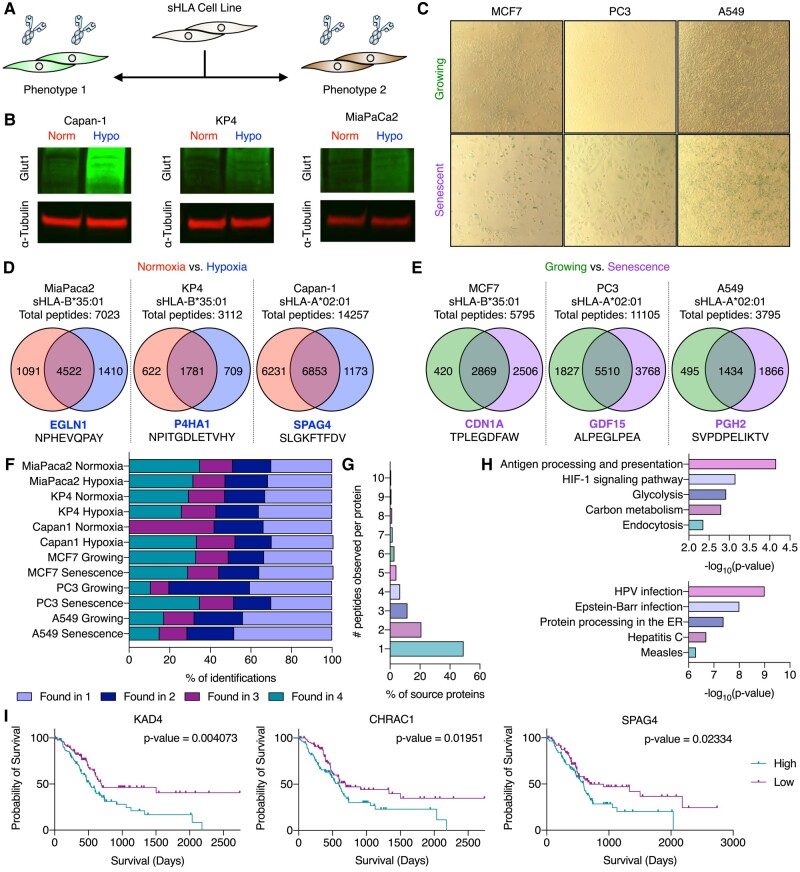
Immunopeptidomics of hypoxic and senescent sHLA cell lines. A) Strategy for identifying phenotype-specific HLA-associated peptides. B) Western blot for Glut1 expression in sHLA cell line samples under normoxic and hypoxic conditions. C) β-Galactosidase staining of sHLA cell line samples. D and E) Venn diagrams of identified peptides across sHLA cell lines across all replicates (*n* = 4, *n* = 3 for CAPAN-1 Normoxia) with a highlighted high-confidence peptide found to be phenotype-specific from each cell line. F) Reproducibility of peptide identification for each sHLA cell line under each condition across all replicates. G) Number of peptides identified per protein in CAPAN-1 normoxic samples. H) GO-term enrichment of select biological processes in high-confidence peptides for hypoxia (top) and senescence (bottom) datasets. I) Survival curves for proteins of which high-confidence hypoxia-specific peptides were identified from sHLA CAPAN-1 cell lines.

Hypoxia and cellular senescence are two disease phenotypes with unexplored immunopeptidomes. The three cell lines commonly used in studying these phenotypes were transduced with our sHLA-A*02:01 or sHLA-B*35:01 constructs. Each cell line was induced into the phenotype of interest prior to doxycycline treatment and subsequent sHLA harvest (Fig. 2A). Establishment of the hypoxic phenotype was confirmed by Glut1 expression (Fig. 2B) and the senescence phenotype was confirmed by β-galactosidase activity staining (Fig. 2C).

Across normoxic and hypoxic samples, peptide identifications ranged from ∼600 to 10,000 per sample across the different phenotypes, with Capan-1 normoxic samples yielding nearly 10,000 HLA-A*02:01-associated peptides from a ∼130 million cell sample (Fig. 2D). This proteomic depth demonstrates the broad applicability of the method toward profiling the immunopeptidome of a single allele. We identified 99, 61, and 135 peptides unique to hypoxia with high confidence (defined as appearing in at least 3 out of 4 biological replicate samples) in MiaPaCa-2, KP4, and Capan-1 cells, respectively. A number of these hypoxia-specific peptides, such as EGLN1 (HLA-B*35:01: NPHEVQPAY), are known to interact with or be driven by the canonical hypoxia-inducible transcription factors (HIF, Fig. 2D) (15). From these high-confidence hypoxia-associated peptides, we identified peptides from several biomarkers of hypoxia, including KAD4 (HLA-A*02:01: NLDFNPPHV) (16). Interestingly, high-confidence peptides, such as the HLA-A*02:01-associated KLHGGILRI derived from EGLN3, were found exclusively in hypoxic samples despite other peptides from the same protein being identified in normoxic samples, which could suggest hypoxia-driven processing.

Of the peptides identified exclusively in senescence within each cell line, 466 in MCF7, 1021 in PC3, and 190 in A549 were found with high confidence. Among these peptides were some from senescence-associated secretory phenotype biomarkers, including GDF15 (HLA-A*02:01: ALPEGLPEA) (17), and from the senescence marker p21 (CDN1A, HLA-B*35:01: TPLEGDFAW) (18), further showcasing the ability of the method to isolate phenotype-relevant peptides (Fig. 2E). Remarkably, these datasets for MCF7 senescent samples (with an average peptide ID count of ∼3,200) were collected using less than 17 million cells, highlighting the potential use of this method with smaller samples. We saw a reproducible increase in peptide identifications across senescent samples compared with their growing counterparts. As senescent cells have an active secretory phenotype, our sHLA construct may be advantageous and have improved trafficking in phenotypes with increased secretory activity.

Our sHLA technology demonstrates high reproducibility, with most peptides identified in at least two biological replicates per condition (Fig. 2F). Mostly, identified peptides were the only derivatives from a given protein sequence, emphasizing the need for reproducible datasets for increased confidence (Fig. 2G). Focusing on high-confidence peptides exclusive to either hypoxic or senescent datasets, we identified processes that mirror the biological disturbance (Fig. 2H). These high-confidence peptides not only enrich for disease-associated processes but could also serve as potential biomarkers (Fig. 2I). In total, our method identified over 30,000 peptides across all cell lines, with >9,000 new to the Immune Epitope Database.

## Discussion

Our sHLA-Fc fusion technology offers advantages for immunopeptidome profiling, including reduced sample input over current sHLA methods, fast preparation, and high reproducibility. The allele-defined sHLA construct avoids the complex samples of traditional pan-MHC immunoprecipitations. The doxycycline-induction feature allows profiling specific cellular states, enabling complex and multicellular models that would be difficult with current immunopeptidomic methods. However, there are limitations. Lentiviral transduction of the sHLA construct may not be suitable for primary or patient-derived cells. The variability in sHLA expression across different conditions currently prevents the absolute or relative quantification of peptide levels possible with membrane-bound HLA samples (19). As such, follow-up confirmation of surface presentation is required. Moreover, the sHLA-Fc fusion might compete with endogenous membrane–bound HLAs for peptide loading, possibly necessitating a knockout of the endogenous allele. In summary, our sHLA-Fc fusion method enables the extraction of HLA-associated peptides in an allele-restricted and phenotype-specific manner. This technology has the potential to accelerate immunopeptidome profiling, enhance prediction algorithms, and propel personalized immunotherapies, aiding in target discovery and peptide triage for antibody-based or T-cell receptor–based therapeutics.

## Materials and methods

### Cloning

sHLA-Fc fusion was cloned into the lentiviral vector pLVX-TetOne-Puro. All constructs were sequence-verified by Sanger sequencing.

### Cell lines and culturing

The B721.221 cells were a generous gift from Dr Lewis Lanier (UCSF). The Capan-1 pancreatic cancer cells were procured from Wells lab frozen stocks. The KP4 and MiaPaCa-2 pancreatic cells were generous gifts from Dr Rushika Perera (UCSF). The A549 lung cancer cells were a gift from Dr Oren Rosenberg (UCSF). The PC3 prostate cancer cells and MCF7 breast cancer cells were purchased from the UCSF Cell Culture Facility. The B721.221, A549, PC3, and MCF7 cells were all grown in RPMI + 10% Tetracycline-negative FBS + 1% Pen/Strep. The Capan-1, KP4, and MiaPaCa-2 cells were all grown in IMDM + 10% tetracycline-negative FBS + 1% Pen/Strep. Cell lines transduced with the sHLA-Fc fusion were cultured in media with 2 μg/mL puromycin. sHLA-Fc fusion cell lines were cultured in respective media without FBS and with doxycycline for sample collection. All cells were grown at 37°C, 5% O_2_, unless otherwise stated.

To generate normoxic Capan-1, KP4, and MiaPaCa-2 cells, cells were grown for 3 days in IMDM + 10% tetracycline-negative FBS + 1% pen/strep at 37°C, 5% O_2_ before beginning doxycycline treatment. Hypoxic cells were grown in a hypoxic chamber in IMDM + 10% Tetracycline-negative FBS + 1% pen/strep at 37°C, 1% O_2_ prior to doxycycline treatment. The hypoxic cells were removed from the chamber only to replace media for the appropriate condition and the exchange was conducted as quickly as possible to avoid the onset of the normoxic phenotype.

The A549, MCF7, and PC3 cells were seeded 1 day prior to treatment. The cells were incubated in media containing either 250 nM doxorubicin (Sigma-Aldrich) or the equivalent volume of DMSO for 24 h. Growing samples were treated with doxycycline immediately after DMSO treatment. For the senescent samples, media was replaced and then subsequently replaced every other day for 8 days of postdoxorubicin treatment before doxycycline treatment. The cells were seeded separately for western blot analysis and β-galactosidase activity staining. β-galactosidase activity staining was performed using a Senescence β-Galactosidase Staining Kit (Cell Signaling) following the manufacturer's protocol.

### Lentivirus and cell line generation

HEK293T cells were cultured in DMEM + 10% FBS + 1% pen/strep. The cells were seeded 5 × 10^5^ per well of a 6-well plate a day prior to transfection. Plasmids at the designated concentrations (1.35 μg pCMV delta8.91, 0.165 μg pMD2-G, 1.5 μg sHLA plasmid) were added to OptiMEM media with a 9-μL FuGENE HD Transfection Reagent (Promega) at a 3:1 FuGENE:DNA ratio, incubated for 30 min, and subsequently transfected into the HEK293T cells. The supernatant was harvested and cleared by passing it through a 0.45-μm filter 72 h posttransfection. The cleared supernatant was added to target cells (∼1 million cells) with 8 μg/mL polybrene and centrifuged at 1,000*×g* at 33°C for 3 h. A total of 24 h post transduction, media was replaced with appropriate fresh media. After an additional 24 h, drug selection for stable cell lines was initiated by the addition of 2 μg/mL puromycin and expanded.

To expand the successfully transduced B721.221 cells, live cells were isolated using SepMate-50 (IVD) tubes and Lymphoprep (Stemcell Technologies). For isolation, cell cultures were centrifuged at 300*×g* for 5 min and resuspended in 5 mL of cell culture media. A quantity of 15 mL Lymphoprep was added to each SepMate-50 (IVD) tube, and the 5 mL cell suspension was subsequently added. The tubes were centrifuged at 400*×g* for 10 min, and then the supernatant was quickly decanted into 30 mL cell media and the SepMate-50 (IVD) tube was discarded. The cell culture was spun at 300*×g* for 5 min and the supernatant was removed. Pellets were resuspended in cell media containing the appropriate drug and expanded. A total of two isolations occurred for each cell line.

### Sample preparation

Cell phenotypes were induced to produce the sHLA-Fc fusions as described. The cells were cultured in media with 2 μg/mL doxycycline for 24 h, and then subsequently cultured in serum-free media with 2 μg/mL doxycycline for 28 h prior to media collection. For each 50 mL of media sample, 100 μL of Pierce Protein A Magnetic beads (Thermo Scientific) were washed twice with PBS prior to use. Beads were added to media—which has previously been filtered with 0.45-μm filters—and rotated for 1 h at 4°C. Samples were spun at 500*×g* for 5 min and media removed. The beads were washed strenuously with 10 mM Tris pH 8.0 made with Optima LC/MS water (Thermo Scientific). After washing, protein/peptides were eluted by incubating beads with 10% acetic acid for 10 min at room temperature. The beads were washed twice with 10% acetic acid, and washes and elution were pooled together. The samples were dried in a Genevac prior to desalting.

The dried-down samples were resuspended in 75 μL of 1% TFA and vortexed vigorously. The samples were centrifuged at 21,000*×g* for 5 min at RT to remove any remaining precipitate. The sample was placed in a magnetic rack, after which the supernatant was removed gently from the tube and placed in a prepared Pierce C18 column according to the manufacturer's instruction. Shortly, each column was washed with 200 μL of 70% acetonitrile in water and spun down at 1,500× *g* until they dried. The columns were further washed with 200 μL of 50% acetonitrile in water and spun till they dried. Following the prewash steps, each column was further washed twice with 200 μL of 5% acetonitrile/0.5% TFA in water and spun till they dried. The sample was then loaded onto the column and spun till it dried. Each sample was reloaded onto the column to maximize peptide yield. The samples were then washed with 2× 200 μL of 5% acetonitrile/0.5% TFA in water, 200 μL of 5% acetonitrile/1% FA in water, and eluted in 2 × 50 μL of 70% acetonitrile in water. The samples were dried to completion.

### Mass spectrometry

LC-MS/MS was performed as previously described (20). Briefly, each sample was brought up in 6.5 μL of 2% acetonitrile/0.1% formic acid in water, vortexed vigorously, and spun down at maximum speed to remove any precipitate. The sample was transferred and 6 μL of the peptide supernatant was separated using a nanoElute UHPLC system (Bruker) with a prepacked 25 cm × 75 μm Aurora Series UHPLC column + CaptiveSpray insert (CSI) column (120 A pore size, IonOpticks, AUR2-25075C18A-CSI) and analyzed on a timsTOF Pro (Bruker) mass spectrometer. The peptides were separated using a linear gradient of 7–30% (Solvent A: 2% acetonitrile, 0.1% formic acid, solvent B: acetonitrile, 0.1% formic acid) over 60 min at 400 nL/min. The data-dependent acquisition process was performed with parallel accumulation-serial fragmentation (PASEF) and trapped ion mobility spectrometry (TIMS) enabled with 10 PASEF scans per top N acquisition cycles. The TIMS analyzer was operated at fixed duty cycles close to 100% using equal accumulation and ramp times of 100 ms each. Singly charged precursors below 800 *m*/*z* were excluded by their position in the *m*/*z*-ion mobility plane, and precursors that reached a target value of 20,000 arbitrary units were dynamically excluded for 0.4 min. The quadrupole isolation width was set to 2 *m*/*z* for *m*/*z* < 700 and 3 *m*/*z* for *m*/*z* > 700 and a mass scan range of 100–1,800 *m*/*z*. TIMS elution voltages were calibrated linearly to obtain the reduced ion mobility coefficients (1/K0) using three Agilent ESI-L Tuning Mix ions (*m*/*z* 622, 922, and 1,222).

### Data analysis

Briefly, for general database searching, peptides for each individual dataset were searched using PEAKS Online X version 1.5 against the entire Swiss-prot Human Proteome (Swiss-prot). Enzyme specificity was set to Unspecific. Peptide length was specified between 8 and 12 amino acids. No fixed modifications were set, while acetylation (N-term) and methionine oxidation were set as variable modifications. The precursor mass error tolerance was set to 20 PPM and the fragment mass error tolerance was set to 0.03 Da. Data were filtered at 1% for both protein and peptide FDR. All MS database searching was based off of four biological replicates. Biological replicates underwent preparation, washing, and downstream LC-MS/MS preparation separately. ICE logos were generated using the server https://iomics.ugent.be/icelogoserver/. Novel peptide IDs were determined by cross-referencing the Immune Epitope Database (as of 2021 October 10).

### Western blot

sHLA-Fc fusion samples from the B721.221 cells were generated and purified as described. Beads were washed three times with PBS and protein was eluted with 0.1 M acetic acid. For growing and senescent samples, the cells were washed twice on plate with PBS prior to lysis. The lysis buffer contained 1 × RIPA (EMD Millipore), 1% protease inhibitor cocktail (Sigma-Aldrich), and 1 mM EDTA. The cells were lysed for 20 min on ice prior to sonication (1 min, 20% amp, 1 s on/off pulse). The cells were spun at 16,000*×g* at 4°C for 5 min, and lysate protein concentration was determined using a Pierce BCA Protein Assay (Thermo Scientific). For hypoxic and normoxic samples, the cells were washed twice on a plate with PBS prior to being dissociated by the addition of versene (PBS + 0.05% EDTA). The cells were lysed using a lysis buffer containing 1 × RIPA (EMD Millipore), 1% protease inhibitor cocktail (Sigma-Aldrich), and 1 mM EDTA. The cells were lysed for 20 min on ice prior to sonication (1 min, 20% amp, 1 s on/off pulse). The cells were spun at 16,000*×g* at 4°C for 5 min, and lysate protein concentration was determined using a Pierce BCA Protein Assay (Thermo Scientific). Samples were run on a Bolt 4–12% Bis-Tris gel (Invitrogen) and transferred to a PVDF membrane (Thermo Scientific) using an iBlot (Thermo Scientific). Membranes were blocked in Odyssey Blocking Buffer (TBS; LiCOR) prior to staining. The membranes were stained with primary anti-FLAG (Cell Signaling, 14793S), antihuman HLA-A (Thermo Scientific, PA5-29911), antihuman HLA-B (Proteintech, 17260), antihuman Glut1 (Abcam, ab115730), and antihuman α-tubulin (Sigma-Aldrich, T6199) antibodies in the blocking buffer for 1 h at room temperature or overnight at 4°C. Secondary staining was performed using goat antirabbit IRDye 800CW and goat antirabbit IRDye 680RD antibodies (LiCOR Biosciences) in the blocking buffer for 1 h at room temperature. Membranes were washed with three 5 min washes of TBST between each staining step. The membranes were imaged using an Odyssey CLx (LiCOR Biosciences).

## Supplementary Material

pgad400_Supplementary_Data

## Data Availability

All of the proteomics datasets have been deposited to the ProteomeXchange Consortium (proteomecentral.proteomexchange.org) via the PRIDE partner repository (PXD045796) (21). All scripts used to process data have been posted on github/nrettko/sHLA-Data-Analysis-Script. All processed data files are included in SI Appendix.
